# Factors Associated With Breast Self‐Examination Among Female Healthcare Providers in Moshi Municipality, Northern Tanzania

**DOI:** 10.1155/ogi/6473357

**Published:** 2026-09-11

**Authors:** Sarah Anga, Avelin Silayo, Rahma Ally, Sebastian Mshilu, William Nkenguye

**Affiliations:** ^1^ School of Medicine, KCMC University, P.O. Box 2240, Moshi, Kilimanjaro, Tanzania, kcmuco.ac.tz; ^2^ Department of Epidemiology and Biostatistics, KCMC University School of Public Health, Moshi, Kilimanjaro, Tanzania; ^3^ Global Emergency Medicine Innovation and Implementation Research Center, Durham, North Caloline, USA

**Keywords:** breast cancer, breast self-examination, female healthcare providers, Tanzania

## Abstract

**Background:**

Breast self‐examination (BSE) is a simple, low‐cost, noninvasive, and painless method that can support early detection of breast cancer (BC). In Tanzania, the burden of BC continues to rise, with increasing incidence and a high proportion of cases presenting at advanced stages. The incidence is projected to increase by up to 82% by 2030. Despite ongoing efforts to improve early detection, mortality remains substantial. Female healthcare providers play a critical role in promoting preventive health behaviors and influencing community attitudes. Therefore, adequate knowledge and a positive attitude toward BSE among healthcare providers are essential for improving its practice. This study aimed to determine the prevalence of BSE practice and its associated factors among female healthcare providers in Moshi Municipality, Tanzania.

**Methods:**

A hospital‐based analytical cross‐sectional study was conducted among female healthcare providers who met the eligibility criteria. A simple random sampling technique was used to select both health facilities and participants, yielding a sample size of 301. Data were collected using a pretested structured questionnaire. Analysis was performed using SPSS Version 25. Descriptive statistics, including frequencies and proportions, were used to summarize categorical variables, while means were used for continuous variables. Binary logistic regression was employed to identify factors associated with BSE practice. Adjusted odds ratios (AOR) with 95% confidence intervals (CI) were reported, and a *p* value < 0.05 was considered statistically significant.

**Results:**

The prevalence of BSE practice was 78.7%; however, only 27.2% of participants reported practicing BSE regularly (monthly) as recommended. Knowledge of BSE was the only independent predictor significantly associated with BSE practice (AOR = 7.72, 95% CI: 2.78–21.44). Additionally, having given birth was significantly associated with BSE practice (OR = 0.37, 95% CI: 0.21–0.66).

**Conclusion:**

Although the overall prevalence of BSE practice among female healthcare providers in Moshi is relatively high, regular practice remains low. Knowledge was the key factor influencing BSE practice. Interventions such as continuous training, reminder systems, and integration of BSE education into workplace health programs are needed to promote consistent and effective practice.

## 1. Introduction

Breast self‐examination (BSE) is a simple, noninvasive, and cost‐effective preventive practice that involves visual inspection and palpation of the breasts to detect abnormal changes [1]. Regular BSE promotes breast awareness, enabling women to recognize changes early and seek timely medical care [2]. It is recommended that women perform BSE monthly, preferably after menstruation [3]. Although there is limited direct evidence that BSE alone reduces breast cancer (BC) mortality, it remains an important secondary prevention strategy in low‐resource settings where access to screening technologies is limited [4]. When combined with clinical breast examination and mammography, BSE contributes to early detection and improved outcomes [5].

BC is a major global public health concern, with increasing incidence and mortality worldwide. It is the leading cause of cancer incidence in 159 out of 185 countries and the leading cause of cancer‐related deaths in 110 countries, accounting for approximately one in four cancer cases and one in six cancer deaths among women globally [2, 6, 7]. The burden is rising in developing countries, partly due to urbanization and adoption of lifestyle‐related risk factors [8]. In sub‐Saharan Africa, BC is among the leading causes of cancer morbidity and mortality among women, contributing significantly to the region’s cancer burden [9].

In Tanzania, BC is the second most common cancer among women, with an estimated incidence of 23.4 cases per 100,000 women annually. The burden is projected to increase substantially, with expected rises of 82% in incidence and 80% in mortality by 2030 [10]. Late presentation remains a major challenge, as many women seek care at advanced stages due to limited access to screening services, financial constraints, and low awareness [10]. Early detection through practices such as BSE is, therefore, critical in improving outcomes and reducing mortality [10].

Global and national efforts to address BC have focused on comprehensive strategies, including awareness campaigns, early detection, screening, diagnosis, and treatment [11]. The World Health Organization and other international bodies emphasize the importance of empowering women with knowledge and encouraging early health‐seeking behavior [12]. In Tanzania, initiatives such as the Tanzania Comprehensive Cancer Project (TCCP) have been implemented to strengthen cancer control through healthcare worker training, public awareness, screening, and improved access to treatment [13].

Despite these efforts, the uptake of BSE remains suboptimal. Studies have shown low levels of BSE practice among both the general population and healthcare workers. This is concerning, as healthcare providers play a critical role in promoting preventive health behaviors and influencing community practices [14]. In Tanzania, existing studies indicate limited knowledge and low regular practice of BSE among women, along with the presence of misconceptions and cultural beliefs that hinder early detection and timely care‐seeking [15]. Furthermore, some public health messages around BC may unintentionally create fear rather than promote proactive health behavior [16].

While several studies have focused on the general population, there is limited evidence regarding BSE practice among healthcare providers in Tanzania. Understanding their knowledge, attitudes, and practices (KAPs) is essential, given their role as health educators and advocates. Therefore, this study aims to assess the prevalence of BSE practice and its associated factors among female healthcare providers (FHP) in Moshi Municipality, Tanzania.

## 2. Materials and Methods

### 2.1. Study Design

Hospital‐based analytical cross‐sectional study was used to collect data from nine health facilities (Kilimanjaro Christian Medical Centre [KCMC] hospital, St. Joseph Hospital, Mawenzi Regional Hospital, Pasua Health Center, Majengo Health Center, Siima Health Center, Lancet Laboratory, Care plus Health Centre, and Moshi Health Centre) in Moshi Municipality, April to June 2025. This is because cross‐sectional studies are relatively faster to conduct compared to other study designs, and both exposure and outcomes can be studied at the same time.

### 2.2. Setting

The study was conducted in Moshi Municipality, which is a major urban area in the Kilimanjaro Region, Northern Tanzania. It serves as an administrative and economic center for the region and is well known for its proximity to Mount Kilimanjaro. Moshi Municipality is divided into 21 wards with a coverage area of approximately 58 km^2^ [17]. According to the 2022 census, Moshi Municipality has a population of 221,000 people from different ethnic backgrounds and significant migration from rural areas. Moshi Municipality hosts several major public and private healthcare facilities, including KCMC, Mawenzi Regional Referral Hospital, and St. Joseph Hospital, which serve as referral centers for Northern Tanzania. The presence of these tertiary and secondary healthcare facilities provides a unique setting with relatively greater access to health services and trained healthcare professionals compared with many rural areas of Tanzania. Consequently, the findings of this study should be interpreted within the context of an urban healthcare workforce.

### 2.3. Study Population

All FHP aged 20 years and above working in the health facilities and willing to consent and participate in the study were included. However, FHP working at the facilities and have undergone bilateral mastectomy and those who did not to consent were excluded from this study.

### 2.4. Variables

The dependent variable was BSE practice, defined as whether a woman performs breast examination and has a score equal to or above 5 out of 7 “Yes” or “No” questions [2].

And the independent variables used were factors associated with BSE practice including knowledge, attitude, cultural background, and history of BC in the family as well as sociodemographic characteristics including age, working experience, level of education, marital status, tribe, and religion.

### 2.5. Sample Size and Sampling Technique

The sample size for this study was determined using the single population proportion formula, assuming a 95% confidence level (*Z* = 1.96), a prevalence of BSE practice of 43.5% based on a previous study conducted in Rwanda [2], and a margin of error of 5%.

Health facilities included in the study were selected from eligible health facilities within Moshi Municipality. Eligible FHP working in the selected facilities during the study period were approached and invited to participate after confirming eligibility. Recruitment continued until the required sample size was achieved.

Effect estimates were reported with corresponding 95% confidence intervals to describe the magnitude and precision of the observed associations. Statistical significance was assessed at a two‐sided *p* value of < 0.05.

### 2.6. Data Collection Procedure and Data Collection Tools

The data were collected using a structured self‐administered questionnaire from the selected health facilities including KCMC Hospital, St. Joseph Hospital, and Mawenzi Regional Referral Hospital. The questionnaire, which took approximately 3‐4 min to complete, included both closed‐ended and multiple choice questions designed to capture sociodemographic characteristics, BSE practice, knowledge and attitudes toward BSE, family and obstetrics history, and the frequency and methods of BSE practice. The only outcome was BSE practice, while other variables were considered as dependent variables.

Knowledge of BSE was assessed using a structured questionnaire adapted from previously published studies on BC awareness and BSE practice among healthcare providers and women in sub‐Saharan Africa. The questionnaire comprised *X* knowledge items covering key domains, including BC risk factors, warning signs and symptoms, the purpose of BSE, the recommended timing and frequency of BSE, the appropriate technique for performing BSE, and the importance of early detection. Each correct response was awarded one point, while incorrect or “don’t know” responses received zero points, resulting in a total possible score ranging from 0 to *X*. Participants, who scored at or above the mean/median or predetermined cutoff, were classified as having good knowledge, whereas those scoring below the cutoff were classified as having poor knowledge.

Attitude toward BSE was assessed using *Y* statements adapted from previously validated KAP questionnaires. The statements explored participants’ perceptions of the importance of BSE, confidence in performing BSE, perceived benefits of regular BSE, willingness to practice BSE, and beliefs regarding early detection of BC. Responses were measured using a five‐point Likert scale ranging from “*strongly disagree*” 1 to “*strongly agree*” 5. Negatively worded items were reverse‐coded before analysis to ensure consistency in scoring. Individual item scores were summed to obtain a total attitude score, with higher scores indicating a more positive attitude toward BSE. Participants scoring at or above the mean/median or predetermined cutoff were categorized as having a positive attitude, whereas those scoring below the cutoff were categorized as having a negative attitude.

The questionnaire was adapted from previously published instruments and reviewed by experts in public health and BC prevention to establish content validity. Prior to the main study, the questionnaire was pretested among FHP from a health facility outside the study area to assess clarity, relevance, and comprehensibility. Feedback obtained during the pilot study was incorporated into the final questionnaire before data collection commenced.

Researchers ensured that participants understood the purpose of the study and maintained confidentiality, with informed consent obtained from all participants. Participation was voluntary, and the participants could discontinue participation at any time and for any reason.

### 2.7. Ethical Approval

Ethical review and approval for the study was obtained from KCMC University research and ethical committees with ethical number UG54/2025. Permission letters were sought from administration of selected hospitals and facilities to allow us to conduct the research in their centers. A written informed consent form requested from each participant after being enlightened about the study, and its importance, then full autonomy was granted to make an informed decision regarding their participation. Participants were allowed to withdraw at any time without penalization and the confidentiality to the given information was granted, including use of numbers instead of names.

### 2.8. Data Management and Analysis

The questionnaires were reviewed daily in the field after activities to check if there were errors or mistakes to correct before participants leave the field, then all questionnaires were collected together and counted to check if there is any missing questionnaire to prevent missing data.

Data were entered, cleared, and analyzed using Statistical Package for Social Sciences Version 25. Descriptive statistics was used to summarize data by mean, and standard deviation was used to summarize continuous data, while a proportion was used to summarize categorical data. Prevalence of BSE practice was calculated as percentage of participants who practice BSE out of total participant who participated in the study. Bivariate analysis was used to test the association between BSE practice and independent variables, and multivariate analysis was used to determine factors associated with BSE practice and factors with *p* value < 0.05 considered to be statistically significant. The findings will be presented using tables and graphs.

## 3. Results

### 3.1. Sociodemographic Characteristics of Participants

Half of the FHP in the study were aged 20–29 years, 160 (53.2%) participants, making this the most common age group. Nurses were the largest professional group 130 (34.2%), followed by doctors and others (radiologists). One hundred and thirty‐one (43.5%) participants had diploma and 93 (30.9%) bachelor’s degree, while a few 23 (7.6%) had no formal education. Most participants were Christian 234 (77.7%), and more than half were not married (60.1%) Table 1.

**TABLE 1:** Sociodemographic characteristics of female healthcare providers in Moshi Municipality 2025 (*N* = 301).

Variables	Frequency	Percentage
Age in years		
20–29	160	53.2
30–39	78	25.9
40–49	31	10.3
50>	32	10.6
Profession		
Doctor	58	19.3
Nurse	103	34.2
Midwife	16	5.3
Pharmacists	40	13.3
Laboratory technicians	28	9.3
Others	56	18.6
Level of education		
Bachelor’s degree	93	30.9
Certificate	49	16.3
Diploma	131	43.5
Master’s degree/higher	5	1.7
No formal education	23	7.6
Religion		
Christian	234	77.7
Non‐Christian	67	22.3
Marital status		
Married	120	39.9
Not married	181	60.1

### 3.2. Obstetrics and BC Characteristics of the Participants

Obstetrics and BC characteristics are shown in Table 2. More than half of the participants (53.8%) had been pregnant before, and 52.5% had given birth. When asked about family history of BC, 11% said yes, 71.4% said no, and 17.6% were not sure. Only a few (5.6%) had ever been diagnosed with a breast disease, and the reported diseases were mastitis and lumps.

**TABLE 2:** Obstetrics and breast cancer characteristics (*N* = 301).

Variables	Frequency	Percentage
Ever been pregnant		
Yes	162	53.8
No	139	46.2
Ever given birth		
Yes	158	52.5
No	143	47.5
Family history of breast cancer		
Yes	33	11
No	215	71.4
Not sure	53	17.6
Ever been diagnosed with any breast disease		
Yes	17	5.6
No	284	94.4

### 3.3. Prevalence of BSE Practice

**FIGURE 1:**
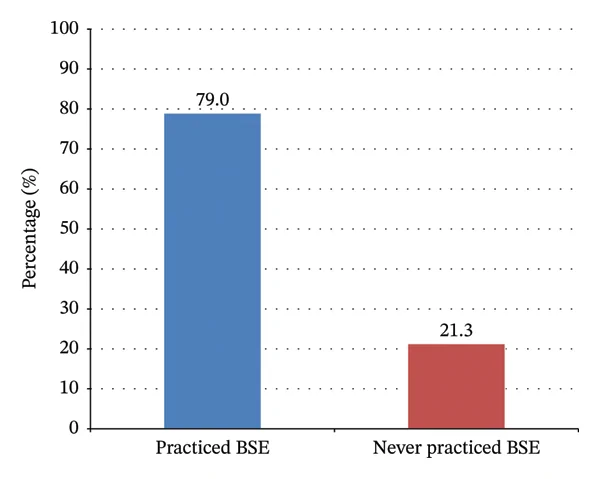
Prevalence of BSE practice.

Out of the 301 FHP surveyed, 78.7% (*n* = 237) reported that they had practiced BSE, while 21.3% (*n* = 64) indicated that they had never practiced, as shown in Figure 1.

### 3.4. Frequency of BSE Practice

**FIGURE 2:**
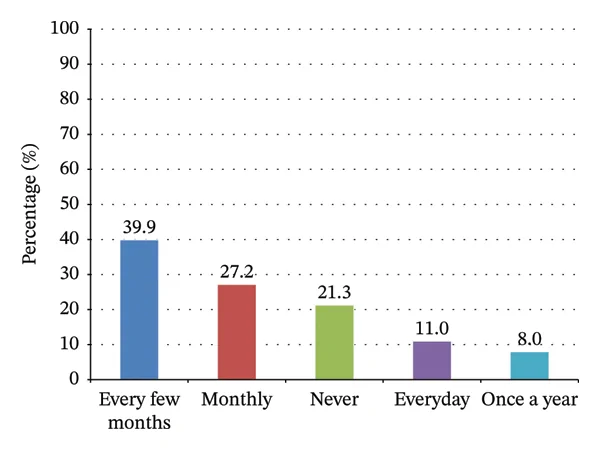
Frequency of breast self‐examination (BSE) practice (*N* = 301).

Regarding the frequency of BSE among the respondents, 39.9% (*n* = 120) reported performing BSE every few months, while 27.2% (*n* = 82) conducted it monthly, aligning with the recommended practice. A smaller proportion, 8.0% (*n* = 24) performed BSE once a year, and 3.7% (*n* = 11) reported practicing it daily. Notably, 21.3% (*n* = 64) of participants indicated they never performed BSE, highlighting a significant gap in regular screening behavior among healthcare providers, as shown in Figure 2.

### 3.5. Techniques of Practicing BSE

Participants reported using a variety of techniques while performing BSE. The most frequently used method was lying down on the bed 61.0% (*n* = 144), followed by nipple squeezing 47.5% (*n* = 112), and using the palm of the three middle fingers 44.5% (*n* = 105). Additionally, 44.1% (*n* = 104) reported following a circular or upward motion, and 42.4% (*n* = 100) conducted BSE in front of a mirror. Less commonly, participants examined the armpit, 30.9% (*n* = 73), or performed BSE while showering, 13.6% (*n* = 32).

Furthermore, 63.12% reported using multiple techniques when performing BSE, indicating a mixed but relatively comprehensive approach among many respondents. The most mixed used method was (in front of the mirror + examine the armpit + follow circular motion + squeeze the nipple).

**FIGURE 3:**
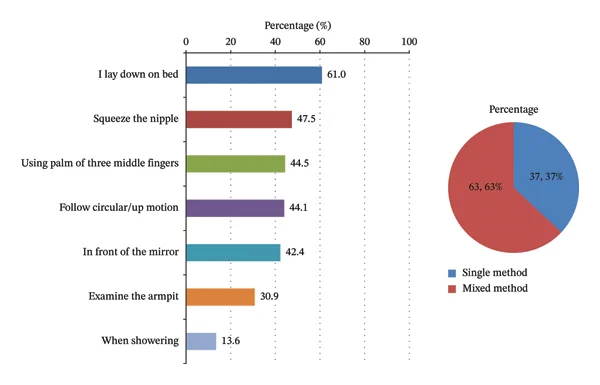
Methods of practicing breast self‐examination (BSE).

These findings suggest that while several standard techniques are being used, the application is inconsistent, and not all participants follow the complete recommended method for effective BSE, as shown in Figure 3.

### 3.6. Common Reasons for Not Performing BSE Regularly

**FIGURE 4:**
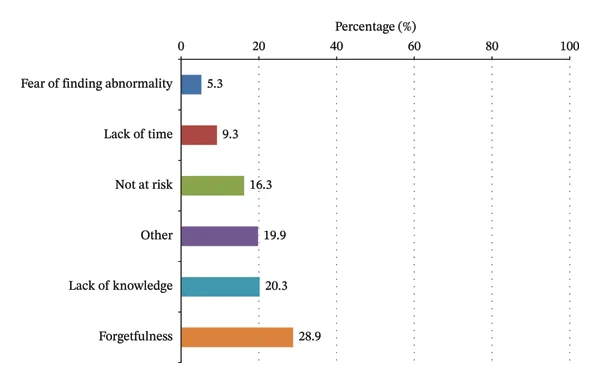
Reasons for not performing BSE regularly.

Out of 301 healthcare providers who stated their reasons for irregular BSE, 28.9% said that they forget to do it, 20.3% lacked knowledge on how or why to perform BSE, and 16.3% believed that they were not at risk. Lack of time accounted for 9.3%, and fear of finding an abnormality for 5.3%. Other reasons, including reliance on health professionals for breast checks and procrastination despite awareness, constituted 19.9%, as shown in Figure 4.

## 4. Factors Influencing Practice of BSE

### 4.1. Sociodemographic Characteristics of the Participants

Association between sociodemographic characteristics of the participants and the practice of BSE is shown in Table 3. Age, profession, education level, religion, and marital status were assessed for their relationship with BSE practice.

**TABLE 3:** Sociodemographic characteristics influencing BSE practice (*N* = 301).

Variable	Total (*N* = 301)	Practice of BSE *n* (%)	OR	95% CI
Age in years				
Above 50	32	23 (71.9)	1	
Below 50	269	213 (79.2)	1.488	0.652–3.396
Profession				
Doctor	58	46 (79.3)	1	
Nurse	103	84 (81.6)	1.153	0.515–2.585
Midwife	16	14 (87.5)	1.826	0.364–9.154
Pharmacists	40	30 (75.0)	0.783	0.301–2.038
Laboratory technicians	28	21 (75.0)	0.783	0.270–2.271
Others	56	41 (73.2)	0.713	0.299–1.699
Level of education				
No formal education	23	20 (87.0)	1	
Master’s degree/higher	5	4 (80.0)	0.6	0.049–7.345
Diploma	131	97 (74.0)	0.428	0.120–1.531
Certificate	49	38 (77.6)	0.518	0.129–2.074
Bachelor’s degree	93	77 (82.8)	0.722	0.191–2.723
Religion				
Non‐Christians	67	56 (83.6)	1	
Christian	234	180 (76.9)	1.358	0.378–4.871
Marital status				
Not married	181	142 (78.5)	1	
Married	120	94 (78.3)	0.813	0.293–2.262

Women of less than 50 years had 1.49 times higher odds of practicing BSE compared to those above 50 years. Midwives had 1.83 higher odds of practicing BSE compared to doctors, while pharmacists, laboratory technicians, and other professionals had lower odds.

Compared to women with no formal education, those with a diploma, certificate, bachelor’s degree, and master’s degree or higher had lower odds of practicing BSE. Christian women had 1.36 times higher odds of practicing BSE compared to non‐Christians (which includes Muslims and pagans). Married women have 0.81 lower odds of practicing BSE compared to unmarried women.

### 4.2. Obstetrics and Cancer History Characteristics

Women who had ever been pregnant had significantly higher odds of practicing BSE compared to those who had never been pregnant. Those who had ever given birth had significantly less odds of practicing BSE compared to those who had never given birth.

Participants with a family history of BC had 1.62 higher odds of practicing BSE compared to those without such history. Women who had ever been diagnosed with a breast disease had 0.47 lower odds of practicing BSE compared to those who had never been diagnosed with any breast disease, as shown in Table 4.

**TABLE 4:** Obstetrics and cancer history characteristics (*N* = 301).

Variable	Total (*N* = 301)	Practice of BSE *n* (%)	OR	95% CI
Ever been pregnant				
Yes	162	140 (86.4)	2.850	1.603–5.069
No	139	96 (69.1)	1	
Ever given birth				
Yes	158	136 (86.1)	0.376	0.212–0.669
No	143	100 (69.9)	1	
Family history of breast cancer				
Yes	33	28 (84.8)	1.615	0.598–4.365
No	268	208 (77.6)	1	
Ever been diagnosed with any breast disease				
Yes	17	15 (88.2)	0.468	0.104–2.100
No	284	221 (77.8)	1	

### 4.3. Knowledge, Cultural, Religious Factors, and Attitude Influencing BSE Practice

Participants with knowledge of BSE were significantly more likely to practice it than those who had no knowledge, AOR of 7.72 (95% CI: 2.78–21.44), as shown in Table 5.

**TABLE 5:** Knowledge, cultural, religious factors, and attitude influencing BSE practice.

Variable	Total (*N* = 301)	Practice of BSE *n* (%)	AOR	95% CI
Knowledge				
No	19	7 (36.8)	1	
Yes	282	229 (81.2)	**7.718**	**2.779–21.435**
Cultural beliefs				
No	248	196 (79.0)	1	
Yes	53	40 (75.5)	1.400	0.0744–2.634
Religious beliefs				
No	174	132 (75.9)	1	
Yes	127	104 (81.9)	0.648	0.298–1.408
Attitude				
Poor	43	33 (76.7)	1	
Good	258	203 (78.7)	0.987	0.440–2.214

*Note:* Bold values are an estimate with *p* value of less than 0.05.

## 5. Discussion

The present study assessed the prevalence of BSE practice and its associated factors among FHP in selected health facilities in Moshi Municipality, Tanzania. Several important findings emerged. Although a high proportion of participants (78.7%) reported ever practicing BSE, only 27.2% reported regular practice, indicating a substantial gap between awareness and consistent preventive behavior. Knowledge was the only statistically significant predictor of BSE practice, while factors such as education level and family history of BC also demonstrated meaningful associations. Additionally, forgetfulness emerged as the most commonly reported barrier to BSE practice.

The high proportion of participants who had ever practiced BSE reflects a generally good level of awareness among healthcare providers, which is expected given their medical background and exposure to health information. However, the low prevalence of regular practice highlights a critical gap between knowledge and behavior. This finding suggests that awareness alone may not be sufficient to sustain consistent preventive practices, even among healthcare professionals. Similar patterns have been reported in other settings, including Turkey, where high levels of BSE awareness did not translate into regular practice [18]. This discrepancy may be explained by competing clinical responsibilities, perceived low personal risk, or lack of structured reminders, all of which can influence adherence to preventive behaviors.

These findings can also be interpreted within the Health Belief Model, which proposes that preventive health behaviors are influenced by perceived susceptibility, perceived benefits, perceived barriers, self‐efficacy, and cues to action. Although healthcare providers possess substantial knowledge regarding BC, competing priorities and the absence of regular reminders may reduce cues to action, thereby limiting consistent BSE practice despite adequate awareness.

The prevalence of regular BSE practice in this study (27.2%) was slightly lower than that reported in Rwanda (33%) and Ethiopia (32.6%) [2, 19]. These differences may reflect variations in institutional emphasis on preventive care, access to continuous professional education, and differences in health promotion strategies across settings. It is also possible that contextual factors such as workload, health system priorities, and cultural perceptions of BC influence the consistency of BSE practice.

Forgetfulness was identified as the most common barrier to BSE (28.9%), followed by lack of knowledge (20.3%), while fear of detecting abnormalities was the least reported reason. This finding suggests that, among healthcare providers, practical and behavioral factors may be more influential barriers than psychological ones. In contrast, studies from Rwanda and Ethiopia reported fear of diagnosis as a leading barrier [2, 19], highlighting important contextual differences in perception and health‐seeking behavior. The prominence of forgetfulness in this study underscores the potential value of simple behavioral interventions, such as reminders or integration of BSE into routine self‐care practices.

Within the Health Belief Model, forgetfulness may represent a lack of cues to action rather than inadequate knowledge. This suggests that interventions such as workplace reminder systems, periodic educational reinforcement, mobile phone reminders, and integration of BSE into routine occupational health programs may improve adherence to regular BSE practice among healthcare providers.

Knowledge was found to be a key determinant of BSE practice, with 81.2% of participants who practiced BSE demonstrating good knowledge. This aligns with the findings from studies in Iran and other settings, which consistently show that higher knowledge levels are associated with increased engagement in preventive health behaviors [20, 21]. From a behavioral perspective, knowledge enhances perceived benefits and self‐efficacy, both of which are critical components of health behavior change models. This highlights the continued importance of targeted education and reinforcement, even among healthcare providers.

Interestingly, participants with lower levels of formal education were more likely to practice BSE, a finding also reported in studies from Iran [22]. This counterintuitive result may reflect differences in risk perception and health beliefs. Individuals with higher education levels may rely more on clinical screening methods or may question the effectiveness of BSE, whereas those with lower educational levels may adopt BSE as a more accessible preventive measure. This finding underscores the complexity of health behavior and the need to consider cognitive and perceptual factors beyond formal education.

Alternatively, this finding may reflect residual confounding or the influence of unmeasured factors such as age, professional cadre, years of clinical experience, or workplace responsibilities. Because this study employed a cross‐sectional design, causal relationships cannot be established, and the observed association should, therefore, be interpreted cautiously. Future qualitative and longitudinal studies are warranted to better understand the behavioral and contextual factors underlying this unexpected finding.

Furthermore, individuals with a family history of BC were 1.6 times more likely to practice BSE, consistent with the findings from Iran and Ethiopia [23, 24]. This is likely due to increased perceived susceptibility and heightened awareness of risk, which are well‐established drivers of preventive health behavior. Individuals with direct exposure to disease within their families may be more motivated to engage in early detection practices.

This observation further supports the Health Belief Model, whereby individuals with a family history of BC perceive themselves to be at greater personal risk, thereby increasing their motivation to engage in preventive behaviors such as BSE.

Although BSE remains a widely promoted strategy for increasing breast awareness, particularly in resource‐limited settings, current evidence does not support BSE as a population‐based screening method for reducing BC mortality. The World Health Organization recommends breast awareness and clinical breast examination, where appropriate, as more suitable approaches in low‐resource settings. Therefore, our findings should be interpreted within this context, emphasizing BSE as a means of promoting breast awareness and encouraging timely healthcare seeking rather than as a substitute for established BC screening strategies.

Overall, these findings highlight that while awareness of BSE is relatively high among healthcare providers, consistent practice remains suboptimal. This suggests that interventions should move beyond knowledge dissemination to address behavioral and system‐level barriers, such as forgetfulness, workload, and integration of preventive practices into routine care. Strengthening these areas may improve adherence to BSE and enhance early detection efforts in similar settings. Interventions informed by behavioral theories such as the Health Belief Model may be particularly valuable in improving adherence to preventive practices by addressing perceived barriers while strengthening cues to action and self‐efficacy.

### 5.1. Study Limitations

The first was that the participants were asked to report their own BSE practice, which may not always be accurate due to recall and social desirability bias. Second, the cross‐sectional design limits the ability to establish temporal or causal relationships between associated factors and BSE practice. Consequently, the observed associations should be interpreted cautiously. Additionally, the data primarily consisted of quantitative measures and lacked qualitative insights that could provide a deeper understanding of participants’ perceptions and behaviors.

Furthermore, although the achieved sample size provided adequate statistical power to detect the observed associations, it may have been insufficient to detect smaller but clinically meaningful associations or differences within subgroup analyses. Finally, the study was conducted among FHP from selected urban health facilities in Moshi Municipality, which may limit the generalizability of the findings to rural healthcare providers, other regions of Tanzania, or similar low‐resource settings with different healthcare infrastructures.

## 6. Conclusion

This study found that while most FHP in Moshi (78.7%) had practiced BSE, only a small number (27.2%) did it regularly as recommended. Forgetfulness was the main reason for not practicing, followed by lack of knowledge. Knowledge was the most important factor influencing BSE practice, and having a family history of BC also increased the likelihood of practicing it. These findings highlight the need for improved education and support to encourage regular BSE among healthcare providers. Although BSE should not be considered a replacement for evidence‐based BC screening strategies, it remains an important approach for promoting breast awareness and encouraging timely healthcare seeking in resource‐limited settings. Interventions that improve knowledge while addressing behavioral barriers through reminder systems, workplace health promotion, and theory‐informed behavior change strategies may enhance regular BSE practice among healthcare providers.

## Funding

The authors received no funding for this work.

## Conflicts of Interest

The authors declare no conflicts of interest.

## Data Availability

The data that support the findings of this study are available on request from the corresponding author. The data are not publicly available due to privacy or ethical restrictions.
